# CD146: a potential therapeutic target for systemic sclerosis

**DOI:** 10.1007/s13238-018-0531-x

**Published:** 2018-04-18

**Authors:** Lingling Zhang, Yongting Luo, Xiao Teng, Zhenzhen Wu, Mengtao Li, Dong Xu, Qian Wang, Fei Wang, Jing Feng, Xiaofeng Zeng, Xiyun Yan

**Affiliations:** 1 Department of Rheumatology and Clinical Immunology, Peking Union Medical College Hospital, Chinese Academy of Medical Sciences and Peking Union Medical College, Key Laboratory of Rheumatology and Clinical Immunology, Ministry of Education, Beijing, 100730 China; 2 Beijing Advanced Innovation Center for Food Nutrition and Human Health, China Agricultural University, Beijing, 100193 China; 3 State Key Laboratory of Cardiovascular Diseases, Fuwai Hospital, National Center for Cardiovascular Diseases, Chinese Academy of Medical Sciences & Peking Union Medical College, Beijing, 100037 China; 4 Key Laboratory of Protein and Peptide Pharmaceuticals, Institute of Biophysics, Chinese Academy of Sciences, Beijing, 100101 China

**Dear Editor,**

Systemic sclerosis (scleroderma, SSc) is a chronic disease of connective tissues, and is clinically characterized by persistent fibrosis in the skin as well as in a variety of organs (Katsumoto et al., 2011). The pathogenesis of SSc is complex and involves vasculopathy, autoimmunity and fibrosis. The hallmarks of late-stage SSc are the excessive secretion and accumulation of extracellular matrix (ECM) by aberrantly activated fibroblasts (myofibroblasts) in the skin and internal organs (Bhattacharyya et al., 2012). Similar to other fibrotic diseases, dysfunction of the affected organs is a common feature and results in high morbidity and significantly increased mortality (Akhmetshina et al., 2012). However, effective anti-fibrotic strategies for the treatment of SSc are not available to date, primarily due to an incomplete understanding of the precise mechanism governing skin fibroblast activation during SSc progression (Distler and Cozzio, 2016). Therefore, identification of novel therapeutic targets for fibrotic-targeted therapy of SSc is of paramount importance.

CD146 was originally identified as a biomarker for metastatic melanoma (Lehmann et al., 1987). Mounting evidence suggests that CD146 plays an important role in the progression of many inflammatory diseases (Dagur and McCoy, 2015; Duan et al., 2013; Luo et al., 2017; Xing et al., 2014). Recent studies suggested that CD146/sCD146 represent a novel biomarker useful for assessing the disease activity of SSc (Ito et al., 2017; Kaspi et al., 2017). Consistently, we found that CD146 is up-regulated on dermal fibroblasts of BLM-induced mice and SSc patients (Fig. S1), and genetic deletion of CD146 attenuated dermal fibrosis, as indicated by decreased skin thickness, collagen content and myofibroblast accumulation (Fig. S2A–C). In addition, we observed decreased type I collagen and fibronectin expression (Fig. S2D–F), and fibroblasts accumulation (Fig. 2G–I) in the skin of CD146-deficient mice. Together, the data from both human and mice indicate that CD146 is a fibrosis-related gene and may be critical for the pathogenesis of dermal fibrosis.

**Figure 1:**
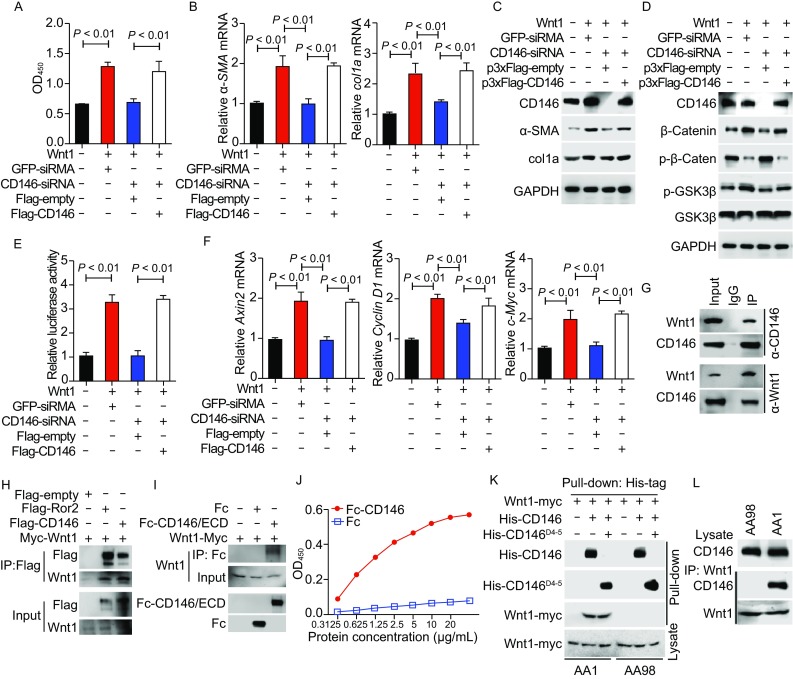
**CD146 is required for fibroblast activation induced by canonical Wnt**. (A) Human skin fibroblasts were transfected with CD146-siRNA and/or Flag-CD146. Cells were subjected to CCK-8 proliferation assays (*n* = 8 per group). (B and C) RT-PCR or Western blot analysis of α-SMA and Col1a1 expression in human skin fibroblasts transfected with CD146-siRNA and/or Flag-CD146. (D) Western blot analysis of β-catenin and GSK3β activation in the presence or absence of Wnt1 in fibroblasts transfected with CD146-siRNA and/or Flag-CD146. (E) TCF luciferase reporter assays to determine the role of CD146 expression in Wnt1/β-catenin activation in human fibroblasts (*n* = 5 per group). (F) RT-PCR analysis of Wnt1-induced expression of β-catenin target genes *axin 2*, *cyclin D1* and *c-Myc* in skin fibroblasts (*n* = 5 per group). (G) Co-IP assays to test the association of endogenous CD146 with Wnt1 in human fibroblast cells. (H) Co-IP assays to test the association of Wnt1 with CD146 or Ror2 in HEK293 cells. Cells were co-transfected with Myc-Wnt1 and Flag-CD146. Proteins were precipitated using anti-Flag mAb M2. (I) Direct interaction between CD146 extracellular domain (CD146/ECD) and Wnt1 as determined by Fc pull-down assay. (J) CD146 binds Wnt1 directly in ELISA. The absorption values at 450 nm were measured. (K) Anti-CD146 AA98 blocked the Wnt1-CD146 interaction in His-tag pull-down assay. (L) The CD146–Wnt1 interaction was blocked by AA98 but not by AA1 as measured in a co-IP assay

As fibroblast accumulation and excessive ECM secretion are the hallmarks of SSc and CD146 elimination reduced the number of skin fibroblasts and inhibited fibrosis, we explored the molecular mechanism behind fibroblast activation. Recently, canonical Wnt signal activation, characterized by increased β-catenin levels, has emerged as a key player in sustained and pathological activation of fibroblasts during the progression of fibrotic diseases (Bergmann and Distler, 2016). Several studies suggest that CD146 expression is potentially linked to Wnt/β-catenin activation in various types of cells (Liu et al., 2012; Tung and Lee, 2017). Therefore, CD146 might facilitate SSc development by mediating Wnt/β-catenin-induced fibrosis.

First, we tested whether CD146 expression was associated with Wnt/β-catenin activation in skin fibroblasts, and found that BLM-induced CD146^−/−^ mice exhibited a decreased number of nuclear β-catenin^+^ fibroblasts (Fig. S3A), decreased expression levels of β-catenin target genes, including *axin 2*, *cyclin D1* and *c-Myc* (Fig. S3B) in lesion skin relative to those in CD146^+/+^ mice. These results imply that CD146 expression is tightly linked to Wnt/β-catenin activation in skin fibroblasts during tissue fibrosis. To investigate the molecular mechanism whereby the absence of CD146 results in reduced fibrogenesis and impaired Wnt/β-catenin activation in skin fibroblasts, we next examined the role of CD146 in Wnt/β-catenin-induced fibroblast activation using primary human skin fibroblasts. Since Wnt1 is the major canonical Wnt ligand specifically up-regulated in fibrotic skin and promotes SSc, we used Wnt1 as a stimulator to trigger fibroblast proliferation and ECM production, mimicking skin fibroblast activation *in vivo*. Results showed that knockdown of CD146 eliminated Wnt1-induced proliferation (Fig. 1A) and the expression of fibrotic genes (α-SMA and type I collagen) (Fig. 1B and 1C), while rescuing CD146 expression restored these functions, demonstrating that CD146 is essential for Wnt1-induced fibroblast proliferation and ECM production.

Then, we examined the role of CD146 in β-catenin activation and the expression of its downstream effectors. We found that Wnt1-induced GSK3β phosphorylation and β-catenin accumulation was impaired by CD146 knockdown and was rescued after restoration of CD146 expression (Fig. 1D). The requirement of CD146 in Wnt/β-catenin activation was further supported by the results of β-catenin/TCF transcription activity assay (Fig. 1E). To establish whether CD146 is required for the transcription of downstream β-catenin target genes upon Wnt1 treatment, we measured the expression levels of *axin 2*, *cyclin D1* and *c-Myc* using RT-PCR (Fig. 1F). These results suggest that CD146 is essential for Wnt1-induced transcription of β-catenin target genes. Together, these observations support the assumption that CD146 is involved in Wnt1-induced β-catenin activation. In order to confirm these results, we isolated primary fibroblasts from the skins of CD146^+/+^ and CD146^−/−^ mice. Identification experiments showed the high purity of the skin fibroblasts (Fig. S4). Consistently, we found that Wnt1 triggered β-catenin activation, fibroblast proliferation, and fibrotic gene expression was inhibited in CD146^−/−^ fibroblasts (Fig. S5). Moreover, using immunoprecipitation and ELISA assays, we found that both CD146 and Wnt1 were immunoprecipitated either by anti-Wnt1 or anti-CD146 antibody (AA1), but not mIgG (Fig. 1G), suggesting that these two molecules were associated in fibroblasts. To confirm the specific interaction, we co-transfected CD146- and Wnt1-expressing plasmids into HEK293T cells, which do not express endogenous CD146 or Wnt1. The reconstitution of CD146/Wnt1 complex suggested a possible association (Fig. 1H). To further investigate whether the binding was direct, we performed *in vitro* pull-down assays using Fc-CD146 and Myc-Wnt1. Our results showed that Myc-Wnt1 bound specifically to Fc-CD146, whereas Fc alone did not show any interaction (Fig. 1I), demonstrating a direct association between CD146 extracellular domain and Wnt1. To further validate the direct interaction, we then performed enzyme-linked immunosorbent assays (ELISA). As shown in Fig. 1J, the binding signal of Wnt1 increased with increasing concentration of Fc-CD146. These results indicate that CD146, as a functional Wnt receptor, promotes Wnt/β-catenin activation in skin fibroblasts, thereby facilitating fibrogenesis.

The above results clearly demonstrated that CD146 plays a pro-fibrotic role and represents a potential therapeutic target for the treatment of dermal fibrosis. We then reasoned that blocking CD146-mediated Wnt/β-catenin activation with a neutralizing antibody would alleviate the fibrotic response. To this end, we first mapped the structural basis of the binding of CD146 to canonical Wnt to domains 4–-5 of CD146 (Fig. 1K). We also screened anti-CD146 antibodies and found that mAb AA98, which specifically recognizes domains 4–-5 of CD146 (Zheng et al., 2009), blocked the interaction of CD146 with canonical Wnt1 ligands (Fig. 1L), thereby inhibit β-catenin activation and the secretion of ECM proteins β-catenin target genes in both human and mouse skin fibroblasts (Figs. S6 and S7). Another anti-CD146 mAb, AA1, which binds to domain 1 of CD146, yielded no comparable effects. Collectively, these results demonstrate that targeting CD146 with AA98 inhibits fibroblast proliferation and ECM production by blocking Wnt/β-catenin activation.

To determine the therapeutic impact of the administration of CD146 blocking antibody AA98 on skin fibrosis *in vivo*, we treated established skin fibrosis model with AA98. Mice were first injected with BLM for 14 days, and antibody treatment was carried out for an additional two weeks (up to day 35) (Fig. 2A). We found that mice administered mIgG or AA1 showed significant increases in dermal thickness and fibrosis compared with those of the mice sacrificed at day 14. In contrast, treatment with AA98 resulted in substantial reductions in dermal thickness (Fig. 2B and 2C), collagen content (Fig. 2D and 2E), ECM production (Fig. 2F–H) and fibroblast content (Fig. 2I–K). We also observed that β-catenin nuclear localization (Fig. S8A) in skin fibroblasts and the expression of β-catenin target genes in lesion skin (Fig. S8B) were significantly reduced in the AA98-treated group. These results indicate that CD146 plays a key role in fibrogenesis by regulating Wnt/β-catenin-mediated fibroblast accumulation and ECM production. Therefore, the neutralization of CD146 with AA98 effectively blocked the further progression of pre-established dermal fibrosis in BLM-induced mouse models. Together, these findings suggest that blocking of CD146 represents a suitable strategy for treating patients affected by progressive dermal fibrosis.

**Figure 2:**
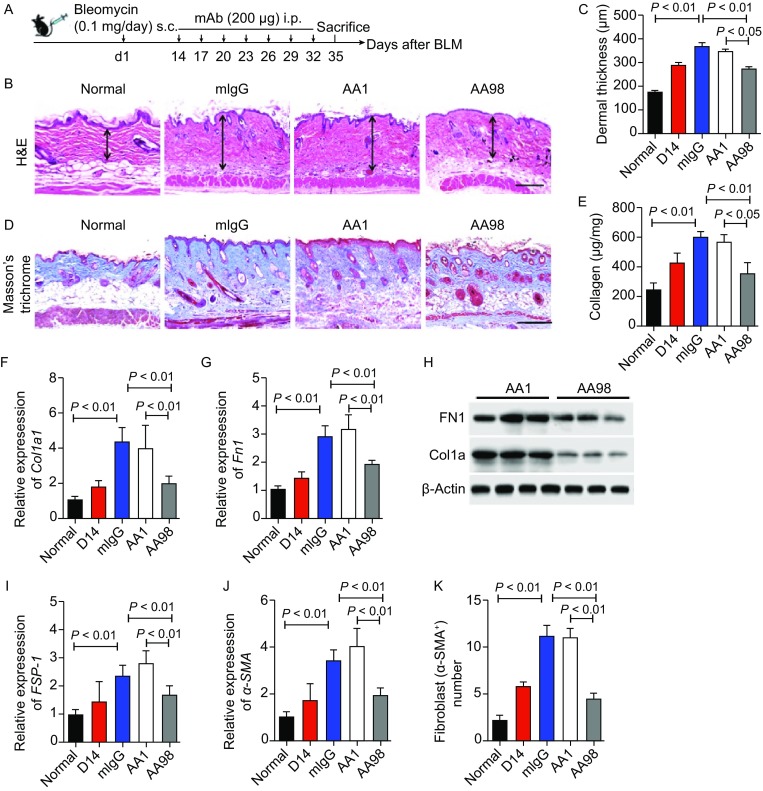
**Therapeutic CD146-blocking with AA98 attenuates BLM-induced skin fibrosis**. (A) Schematic diagram of CD146-targeting therapy using anti-CD146 AA1 or AA98 in BLM-induced skin fibrosis. (B) Mice were treated with AA1 or AA98 during BLM induction for 21 days. Lesion tissues were sectioned and stained, and dermal thickness was measured using Image J. Representative images are shown (scale bar, 100 μm). (C) Quantification of dermal thickness in each group (≥6 sections per mouse, 10 mice per group). (D and E) Skin sections in each group were stained with Masson’s trichrome (D) (scale bar, 100 μm), and collagen content (E) was measured (*n* = 10 per group). (F–H) RT-PCR analysis of *Col1a1* (F) and *Fn1* (G) mRNA expression (*n* = 10 per group) and Western blot analysis of Col1a1 and Fn1 protein expression (H) in fibrotic skin tissues (*n* = 3 per group). (I and J) RT-PCR analysis of *FSP-1* (I) and *α-SMA* (J) mRNA expression in fibrotic skin tissues (*n* = 10 per group). (K) Quantification of α-SMA^+^ fibroblasts in skin tissues (*n* = 10 per group). Significant differences were determined by two-way ANOVA. Error bars indicate mean ± SEM of at least three independent experiments

Current available strategies for SSc treatment are limited, and no anti-fibrotic strategy has been approved for clinical use. The present study provides the first evidence that CD146 plays a pro-fibrotic role by facilitating canonical Wnt-mediated fibroblast activation in the lesion skin, and CD146 might be an efficient therapeutic target for SSc. It is encouraging that the anti-CD146 mAb AA98 showed marked anti-fibrotic effects in BLM-induced SSc, even when applied post-onset of the disease, supporting the notion that CD146 might be an attractive therapeutic target for the treatment of this debilitating disease. As fibrosis-related diseases share common pathological characteristics and core pathways, such as canonical Wnt cascade, these findings should open new avenues for the treatment of other fibrotic diseases, such as pulmonary fibrosis, liver fibrosis, and kidney fibrosis, by targeting CD146.

## Electronic supplementary material

Below is the link to the electronic supplementary material.

Supplementary material 1 (PDF 9906 kb)
