# Supplementary material for: CD146: a potential therapeutic target for systemic sclerosis
Source: Protein Cell. 2018 Apr 18;9(12):1050–4. doi: 10.1007/s13238-018-0531-x (PMC6251808; doi:10.1007/s13238-018-0531-x)
Supplement: Supplementary file 1 — Supplementary material 1 (PDF 9906 kb) [file 13238_2018_531_MOESM1_ESM.pdf]

Supplemental materials

Materials and Methods

Antibodies and reagents

All anti-CD146 antibodies, including mouse anti-CD146 monoclonal antibodies (mAbs) AA98, AA1, and AA4 and rabbit anti-CD146 polyclonal antibody, were generated in our laboratory (Duan et al., 2013). AA98 and AA1 were used for functional assays in parallel. AA1 was used for immunoprecipitation, AA4 for immunohistochemistry, and rat anti-mouse CD146 (Cat# 134701, Clone 9F1; Biolegend, San Diego, CA, USA) for western blotting and immunohistochemistry. Anti-α-SMA mAb (Cat# 19245), anti-Wnt1 (Cat# 2721), anti-p-β-catenin (Cat# 4176), and anti-β-catenin (Cat# 8480) were purchased from Cell Signaling Technology. Anti-collagen1 (Cat# ab34710) and anti-fibronectin (Cat# ab2413) were from Abcam. Recombinant human His-CD146 protein (Cat# 50422-M08H), human Fc-CD146 protein (Cat# 50422-M08H), and Fc protein (Cat #50794-M08H) were obtained from Sino Biological Inc. (Beijing, China). His-$CD146^{D4-5}$ protein was purified in our laboratory.

Cells and transfection

Human skin fibroblasts were obtained from the Cell Resource Center (IBMS, CAMS/PUMC Beijing). Mice dermal fibroblasts were isolated from the dorsal skin tissues of $CD146^{-/-}$ and $CD146^{+/+}$ mice, and cells from passages 3–5 were studied in parallel. Western blot and flow cytometry revealed that these cells expressed fibroblast markers.

For transfection, scrambled non-targeting control siRNA was used as a negative control. Lipofectamine 2000-mediated transfection was employed according to the manufacturer's instructions. To downregulate CD146 expression, 50 nM siRNA targeting CD146 was transfected into human skin fibroblasts. The co-transfection of CD146-siRNA (50 nM) and CD146 expression plasmid (Flag-CD146 plasmid, 2 μg) was performed to restore CD146 expression. The empty vector (Flag-empty) was used as an internal control.

SSc patients and controls

Skin biopsies were obtained from SSc patients and unrelated healthy participants at PUMCH. All SSc patients met the American College of Rheumatology (ACR) criteria for SSc (LeRoy and Medsger, 2001). Patients had been diagnosed with diffuse cutaneous SSc, with the disease duration being less than 5 years in all participants recruited from PUMCH. Healthy participants showed no personal or family history of autoimmune diseases. Skin biopsies were obtained from affected forearm skin tissues, or from a similar location from healthy participants.

Mice, bleomycin (BLM) administration, and antibody treatment

All mice were housed and cared for in a pathogen-free facility at PUMCH. Wild-type (WT) (C57BL/6) mice were purchased from Vital River Laboratory Animal Technology Co., Ltd. (Beijing, China). Both $CD146^{+/+}$ (WT) mice and $CD146^{-/-}$ mice were from a C57BL/6 background. $CD146^{-/-}$ mice were generated using a Cre/*loxP* recombination system by crossing EIIa-cre mice (The Jackson Laboratory, Bar Harbor, ME, USA) with $CD146^{floxed/floxed}$ mice. All genotypes were confirmed by

PCR analysis.

To initiate BLM-induced skin fibrosis, male mice (6–8 weeks of age) were treated by subcutaneous injection with either BLM (0.1 mg/day; Nippon Kayaku Co., Ltd., Tokyo, Japan) dissolved in phosphate-buffered saline (PBS) or PBS alone (n = 10 per group) (Yamamoto et al., 1999). For preventive treatment, male C57BL/6J mice were injected intraperitoneally on days 1, 4, 7, 10, and 13 with anti-CD146 mAb AA98 or AA1 or with the mouse control mIgG (200 μg/mouse). For the disease treatment experiments, the antibody was injected on days 14, 17, 20, 23, 26, 29, and 32 post-BLM administration (the day of fibrosis onset). Mice were sacrificed, and lesioned skin was harvested using an 8-mm skin biopsy punch for analysis.

Histological assessment and immunohistochemistry (IHC)

On the day after the final injection, mice were sacrificed, and skin sections were taken. Sections (5-μm thick) of formalin-fixed paraffin-embedded skin biopsies were stained with hematoxylin and eosin (H&E). To evaluate levels of skin fibrosis in mice treated with BLM, the thickness of the dermis, defined as the distance from the epidermal–dermal junction to the dermal–adipose layer junction, was measured at six randomly selected sites/microscopic fields in each animal (Wu et al., 2012). To analyze the accumulated collagen content in the lesioned skin, sections were stained with Masson's trichrome. Immunohistochemistry was performed on formalin-fixed paraffin-embedded skin biopsies using α-smooth muscle actin (α-SMA).

Collagen measurement

Collagen contents of skin tissues were quantified following the instructions of the

QuickZyme Total Collagen Assay (QuickZyme Biosciences, Leiden, Netherlands). Skin samples from a 6-mm biopsy punch were used (Wu et al., 2014).

RNA isolation and real-time (RT) PCR

Tissue total RNA was isolated using TRIzol reagent (Invitrogen, Carlsbad, CA, USA) and purified with a High Pure RNA Tissue Kit (Roche, Basel, Switzerland). RNA (2 μg) was reverse-transcribed into cDNA. RT-PCR was carried out using SYBR Green PCR Master Mix Plus (Toyobo Co., Osaka, Japan) on an ABI Prism 7000 (Life Technologies, Carlsbad, CA, USA) in triplicate. mRNA levels were normalized to those of the *GAPDH* gene. Relative changes in the levels of genes of interest were determined by the $2^{-\Delta\Delta CT}$ method. Dissociation curves were obtained for each primer pair following PCR to verify the specificity of the primers. The specific primers used for RT-PCR are provided in Supplementary Table 1.

Immunoprecipitation

Cells were lysed with RIPA buffer [150 mM NaCl, 50 mM Tris (pH 8.0), 0.1% sodium dodecyl sulfate (SDS), 0.5% deoxycholate, 1% NP-40, 1 mM phenylmethanesulfonyl fluoride, and Protease Inhibitor Cocktail (Roche)] for 1 h on ice. After centrifugation, the soluble supernatants were pre-cleared with protein G PLUS-Agarose (Santa Cruz Biotechnology Inc., Dallas, TX, USA). Samples were then immunoprecipitated with specific antibodies and 25 μl of protein G-agarose beads. Protein G-bound immunocomplexes were extensively washed with washing buffer [150 mM NaCl, 50 mM Tris (pH 8.0), 0.1% SDS, 0.5% deoxycholate, 0.1% NP-40, 1 mM phenylmethanesulfonyl fluoride and Protease Inhibitor Cocktails] and

boiled in sample loading buffer for SDS-polyacrylamide gel electrophoresis. Immunoprecipitated proteins were detected by western blot.

***In vitro* Fc pull-down assay**

Fc or Fc-CD146 (0.15 μg) was mixed with Myc-Wnt1 in PBS for 2 h. Then, 25 μl of protein G-agarose beads were added and incubated for 1 h. Proteins bound to the beads were then boiled in sample loading buffer and subjected to immunoblotting.

For antibody inhibition assay, 0.15 μg His-CD146 or His-CD146^{D4-5} was mixed with Myc-Wnt1 in PBS for 2 h in the presence or absence of anti-CD146 mAbs. Anti-Myc or anti-His tagged antibody was used for precipitating the protein complex. Precipitates were then boiled in sample loading buffer and subjected to immunoblotting.

Cell proliferation assays

Wnt1-induced fibroblast proliferation was performed using a CCK-8 Cell Counting Kit. Briefly, the same number of fibroblasts was seeded in each well of a 96-well plate. Anti-CD146 mAb AA98 or AA1 (50 μg/ml) was added 1 h before stimulation with Wnt1 supernatant. Then, 48 h after stimulation, the relative cell number was determined by measuring the optical density (OD) of CCK-8 at 450 nm.

Luciferase reporter assays

Fibroblasts used in the luciferase reporter assay were seeded in 48-well plates and transfected in triplicate with plasmids or siRNAs together with Super TOP-FLASH (Addgene, Cambridge, MA, USA). pRL-TK was used as an internal control, and luciferase activity was determined as described.

Immunofluorescence

For immunofluorescence analysis, sections (5-μm thick) were de-paraffinized and stained with Abs specific for α-SMA (a fibroblast marker), CD31, Wnt1, and CD146, followed by fluorescently labeled secondary antibodies. Nuclei were stained with 4', 6-diamidino-2-phenylindole (DAPI). Samples were examined using a confocal laser scanning microscope (Olympus FV 1000; Tokyo, Japan) with a mounted Olympus IX81 digital camera equipped with a 20×/0.75 numeric aperture objective.

Statistical analysis

All experiments were performed in triplicate, and all summary data are expressed as mean ± SEM. A one- or two-way ANOVA test was used to compare differences between groups in the various experiments. SPSS 11.0 for Windows was used to perform the analyses. Differences with p-values < 0.05 were considered to be statistically significant.

REFERENCES

LeRoy EC, and Medsger TA (2001) Criteria for the classification of early systemic sclerosis. J Rheumatol 28:1573-1576

Wu M, Schneider DJ, Mayes MD, Assassi S, Arnett FC et al (2012) Osteopontin in systemic sclerosis and its role in dermal fibrosis. J Invest Dermatol 132:1605-1614

Wu M, Pedroza M, Lafyatis R, George AT, Mayes MD et al (2014) Identification of cadherin 11 as a mediator of dermal fibrosis and possible role in systemic sclerosis. Arthritis Rheumatol 66:1010-1021

Yamamoto T, Takagawa S, Katayama I, Yamazaki K, Hamazaki Y et al (1999) Animal model of sclerotic skin. I: Local injections of bleomycin induce sclerotic skin mimicking scleroderma. J Invest Dermatol 112:456-462.

Supplementary figures and figure legends

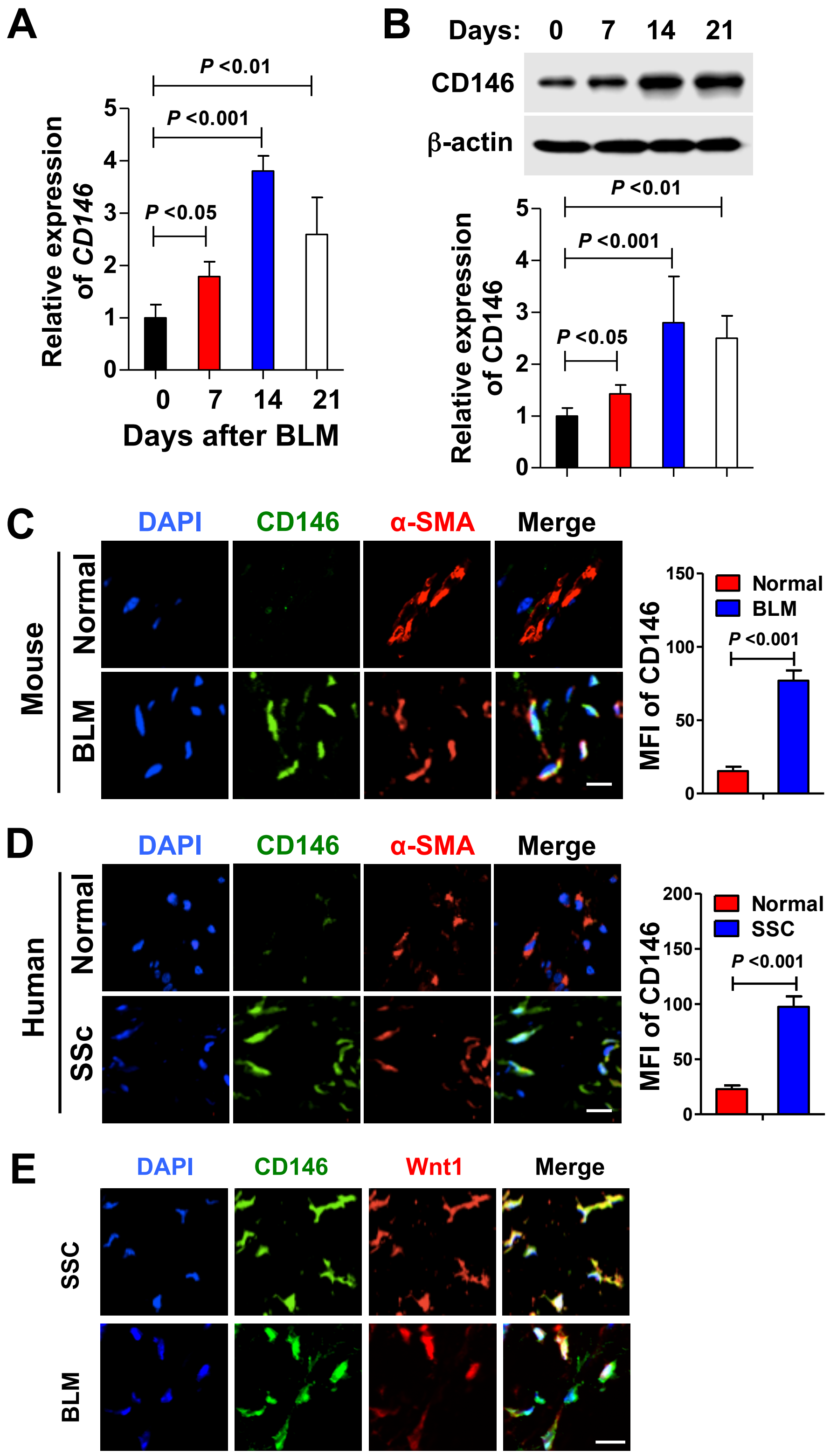

Figure S1. CD146 expression is upregulated in fibroblasts from both BLM-induced mice and SSc patients. (A, B) RT-PCR analysis of *CD146* mRNA

expression (A) and western blot analysis of CD146 protein expression (B) in fibrotic skin tissues of C57BL/6J mice at the indicated time points after BLM induction (n = 5 per group). The lower part of B shows the quantification of CD146 expression. (C) Skin samples from mice with BLM-induced dermal fibrosis or control mice (n = 5 per group) were stained for α-SMA (red), CD146 (green), with co-localization indicated in yellow (right panel). Nuclei were counterstained with DAPI. The mean integral optical density (IOD) of CD146 in α-SMA^{+} fibroblasts was analyzed using Image ProPlus software (n = 5 samples in each group, and at least 5 sections per sample). (D) Skin biopsy samples from SSc patients and healthy participants were stained for α-SMA (red), CD146 (green), with co-localization indicated in yellow (right panel). Scale bars represent 50 μm. (E) Immunohistochemical staining of CD146 (green) and Wnt1 (red) in skin tissues from human SSc patients and BLM-induced mouse models. Nuclei were counterstained with DAPI. Scale bars represent 50 μm. Significant differences were determined by two-way ANOVA. Error bars indicate mean ± SEM of at least three independent experiments.

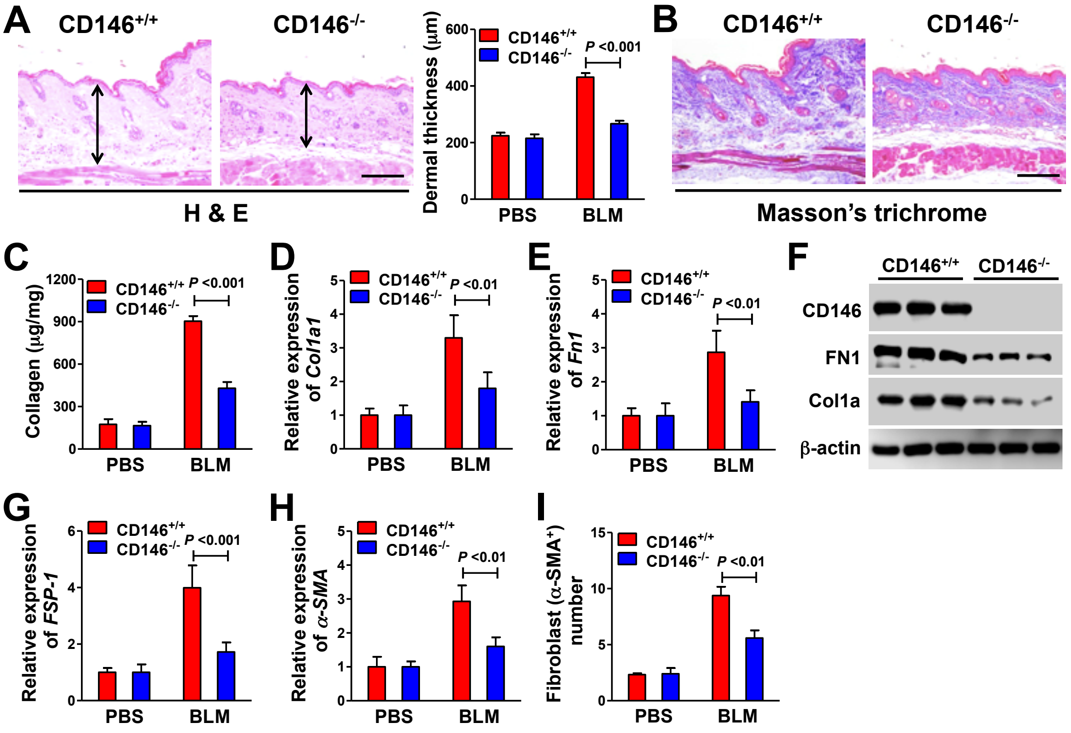

Figure S2. Elimination of CD146 expression attenuates skin fibrosis in BLM-treated mice. (A) CD146$^{+/+}$ and CD146$^{-/-}$ mice were treated with BLM or PBS for 21 days. Lesioned skin tissues were sectioned and subjected to H&E staining, and dermal thickness was measured using Image J software. Representative images are shown (10 mice per group; scale bar, 100 μm). (B, C) Skin sections were stained with Masson's trichrome (B) (scale bar, 100 μm), and collagen content (C) was measured (n = 10 per group). (D–F) RT-PCR analysis of *Col1a1* (D) and fibronectin (*Fn1*; E) mRNA expression (n = 10 per group) or western blot analysis of Col1a1 and Fn1 protein expression (F) in fibrotic skin tissues (n = 3 per group). (G, H) RT-PCR analysis of *FSP-1* (G) and *α-SMA* (H) mRNA expression in fibrotic skin tissues (n = 10 per group). (I) Quantification of α-SMA^{+} fibroblasts in skin tissues (n = 10 per group). Error bars indicate mean ± SEM of at least three independent experiments.

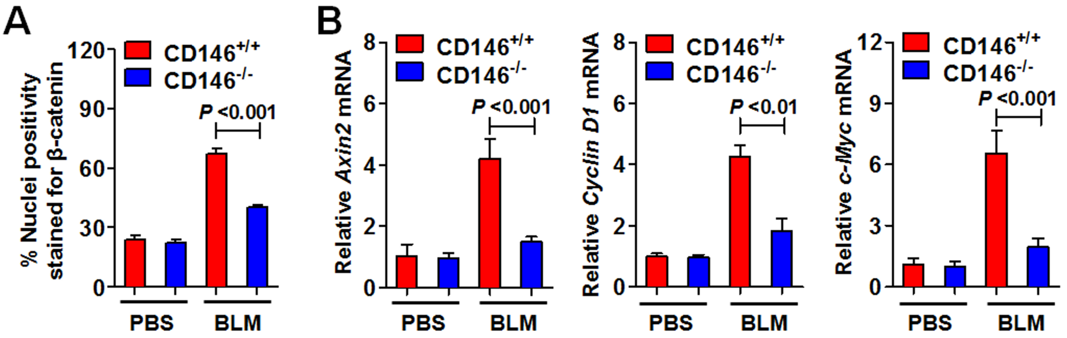

Figure S3. CD146 expression is associated with Wnt/β-catenin activation in skin fibroblasts. (A) Quantification of the number of β-catenin$^+$ fibroblasts in fibrotic skin samples (n = 8 per group). (B) RT-PCR analysis of β-catenin target genes axin 2, cyclin D1, and c-Myc in fibrotic skin tissues (n = 10 per group). Significant differences were determined by two-way ANOVA. Error bars indicate mean ± SEM of at least three independent experiments.

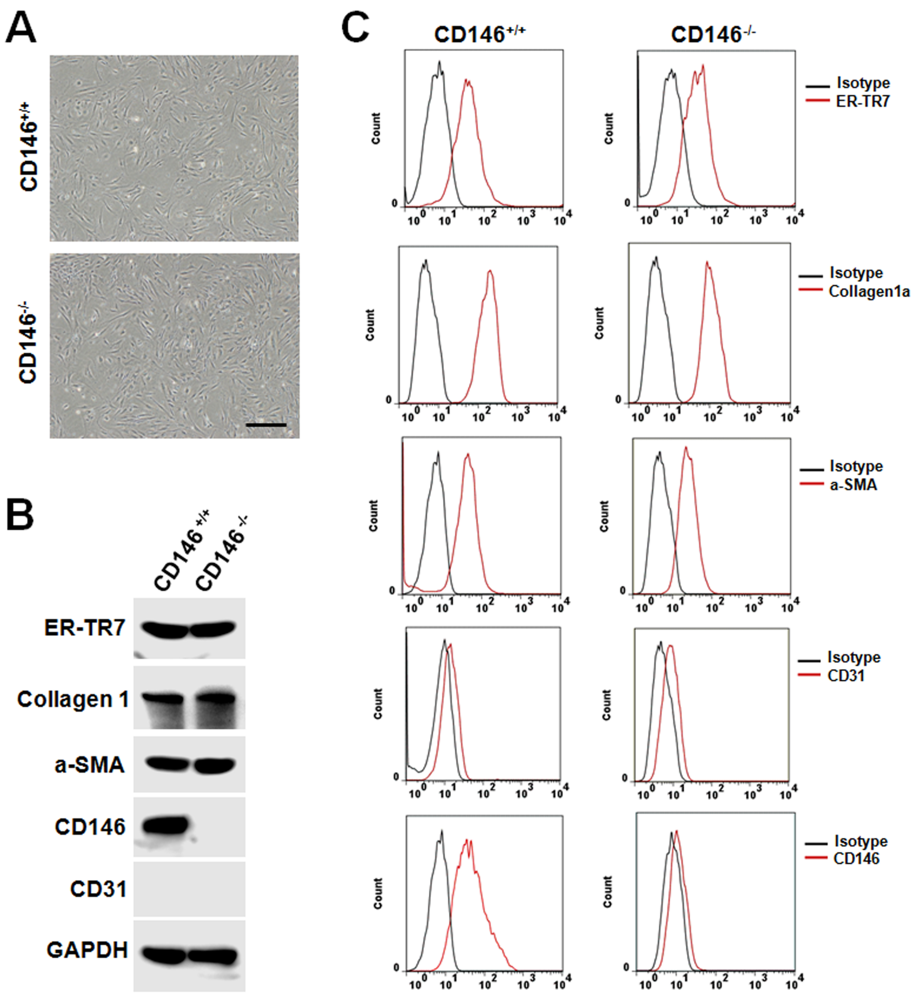

Figure S4. Isolation and characterization of primary fibroblasts from mouse skin. Skin fibroblasts were isolated from CD146$^{+/+}$ or CD146$^{-/-}$ mice. (A) Morphologies of mouse skin fibroblasts isolated from CD146$^{+/+}$ or CD146$^{-/-}$ mice. Scale bars represent 50 μm. (B, C) Expression of fibroblast markers collagen 1 and α-SMA and endothelial marker CD31 on isolated fibroblasts as determined by western blot (B) or flow cytometry analysis (C). All experimental data were verified in at least three independent experiments.

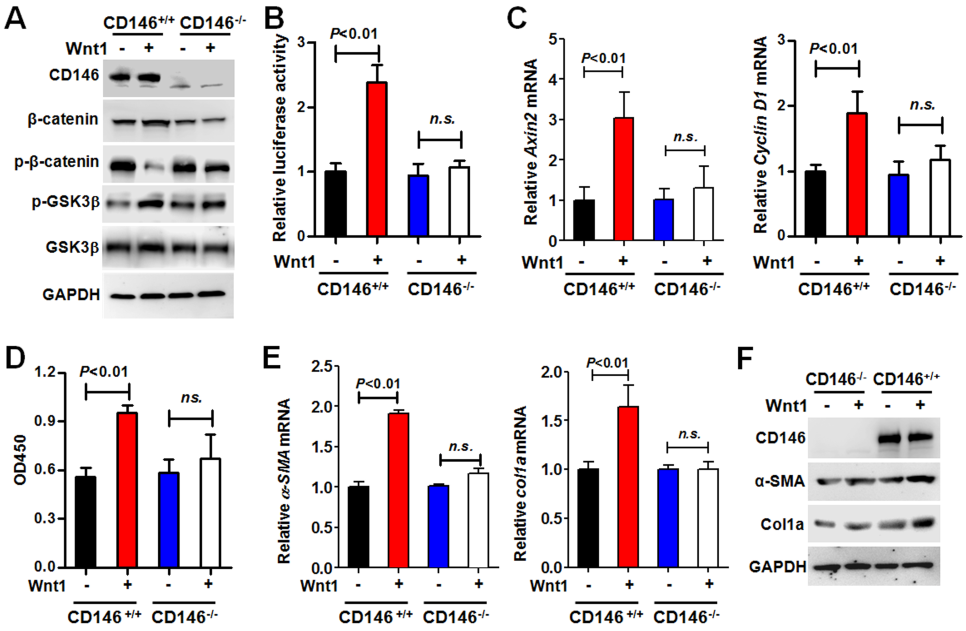

Figure S5. CD146 is required for fibroblast activation induced by canonical Wnt. (A) Skin fibroblasts from CD146$^{+/+}$ or CD146$^{-/-}$ mice were incubated with Wnt1 supernatant for 3 h, and proteins were blotted with their corresponding Abs. Proteins on Western blots were quantified by measuring the band density, which was then normalized to the density of the GAPDH band on the same blot. (B) CD146 is required for Wnt1 signaling as determined by luciferase reporter assays in mouse fibroblasts (n = 8 per group). (C) Skin fibroblasts were incubated with Wnt1 supernatant. Then, the mRNA levels of β-catenin target genes were determined by real-time PCR. (D) Skin fibroblasts from CD146$^{+/+}$ or CD146$^{-/-}$ mice were subjected to proliferation assays (n = 8 per group). (E, F) CD146$^{+/+}$ or CD146$^{-/-}$ mouse skin fibroblasts were stimulated with Wnt1 supernatant for 36 h. The gene and protein expression levels of the fibrotic genes α-SMA and Col1a1 were determined by RT-PCR (E) and western blot (F) (n = 5 per group). Error bars represent the mean ± SEM of three independent experiments. Significant differences were determined by two-way ANOVA.

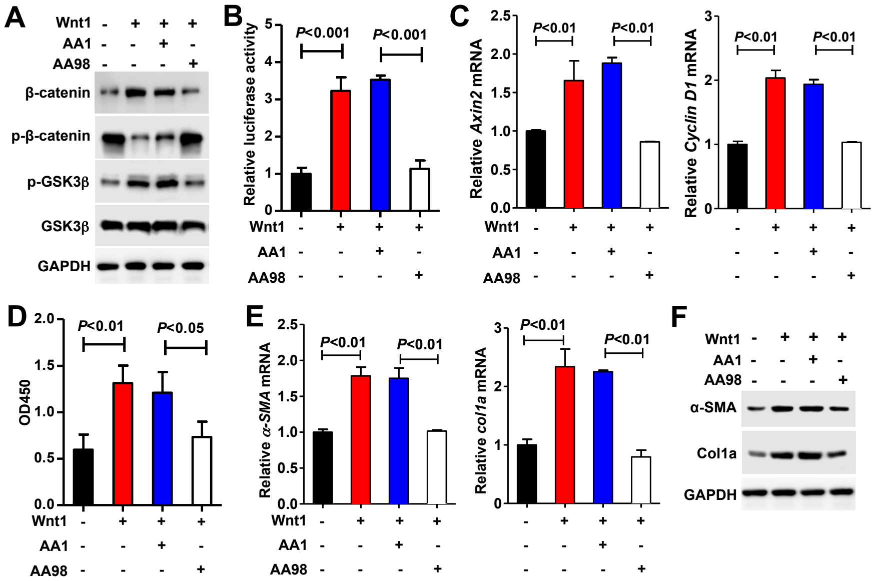

Figure S6. Anti-CD146 AA98 inhibits Wnt/β-catenin activation and human fibroblast proliferation and ECM production. (A) Western blot analysis of Wnt1-induced β-catenin and GSK3 expression in the presence of AA1 or AA98 in human skin fibroblasts. (B) TCF luciferase reporter assays to determine the role of anti-CD146 in Wnt1/β-catenin activation in human fibroblasts (n = 5 per group). (C) RT-PCR analysis of Wnt1-induced expression of β-catenin target genes axin 2, cyclin D1, and c-Myc in skin fibroblasts (n = 5 per group). (D) CCK-8 assays for Wnt1-induced proliferation of human skin fibroblasts in the presence of AA1 or AA98 (n = 8 per group). (E, F) RT-PCR and western blot analysis of α-SMA and Col1a1 mRNA expression (E) or protein expression (F) in human skin fibroblasts in the presence of AA1 or AA98 (n = 5 per group). Significant differences were determined by two-way ANOVA. Error bars indicate mean ± SEM of at least three independent experiments.

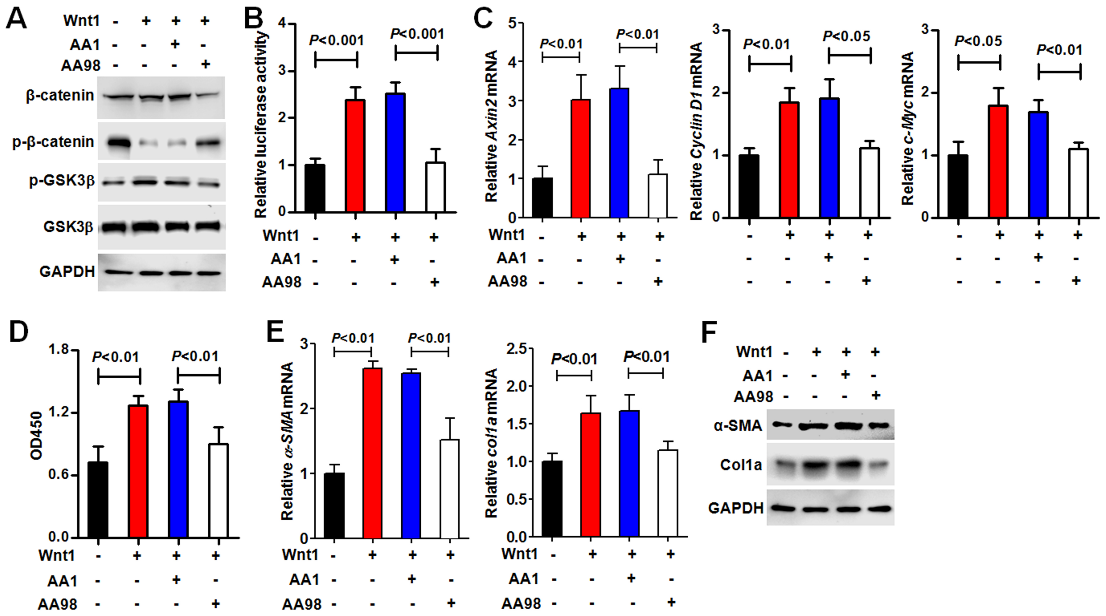

Figure S7. Anti-CD146 AA98 inhibits Wnt/β-catenin activation and mouse fibroblast proliferation and ECM production. (A) Western blot analysis of Wnt1-induced β-catenin and GSK3 expression in the presence of AA1 or AA98 in human skin fibroblasts. (B) TCF luciferase reporter assays to determine the role of anti-CD146 in Wnt1/β-catenin activation in mouse fibroblasts (n = 5 per group). (C) RT-PCR analysis of Wnt1-induced expression of β-catenin target genes axin 2, cyclin D1, and c-Myc in skin fibroblasts (n = 5 per group). (D) CCK-8 assays for Wnt1-induced proliferation of mouse skin fibroblasts in the presence of AA1 or AA98 (n = 8 per group). (E, F) RT-PCR and western blot analysis of α-SMA and Col1a1 mRNA expression (E) or protein expression (F) in mouse skin fibroblasts in the presence of AA1 or AA98 (n = 5 per group). Significant differences were determined by two-way ANOVA. Error bars indicate mean ± SEM of at least three independent experiments.

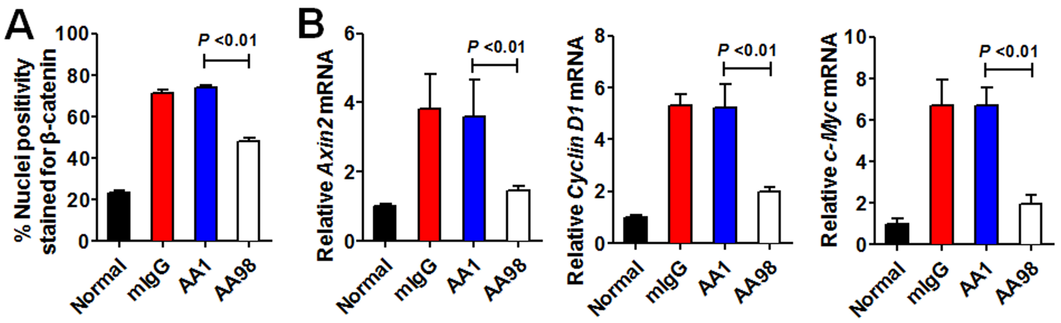

Figure S8. Targeting of CD146 with AA98 inhibits Wnt/β-catenin activation in skin fibroblasts. (A) Quantification of the number of β-catenin^{+} fibroblasts in fibrotic samples in each group (n = 8 per group). (B) RT-PCR analysis of β-catenin target genes axin 2, cyclin D1, and c-Myc in fibrotic skin tissues from each group (n = 10 per group). Significant differences were determined by two-way ANOVA. Error bars indicate mean ± SEM of at least three independent experiments.

Supplementary Table 1. Primers for real-time PCR analysis

Primer	5’ - 3’
mAxin2-f	GCTGAGTGTGTTTGAGAGACTGA
mAxin2-r	TCCCACTTCCAGGTTTAAAAGCA
hAxin2-f	TGAGAGGAACTGGAAGAAGAAGAG
hAxin2-r	AGACATGGGTTTGGTGACCTG
hC-myc-f	CAAGAGGCGAACACACAACG
hC-myc-r	GTCGTTTCCGCAACAAGTCC
mC-myc-f	GTTGGAAACCCCGCAGACAG
mC-myc-r	GTGGGAAGCAGCTCGAATTT
hCyclinD1-f	GAAGTTGCAAAGTCCTGGAGC
hCyclinD1-r	TGGTTTCCACTTCGCAGCA
mActin-f	GAAGTGTGACGTTGACATCCGTA
mActin-r	CTCAGGAGGAGCAATGATCTTGA
mCyclinD1-f	TAGGCCCTCAGCCTCACTC
mCyclinD1-r	CCACCCCTGGGATAAAGCA
mWnt1-f	GAGTGCAAATGGCAATTCCG
mWnt1-r	GCTGCAGTGACAACATCGAT
mCol1a1-f	TTCTCCTGGCAAAGACGGAC
mCol1a1-r	CGGCCACCATCTTGAGACTT
m apha-SMA-f	CCCTGGAGAAGAGCTACGAAC
m apha-SMA-r	CCCCTGACAGGACGTTGTT
